# Assessment of Self-Medication Practices and Its Associated Factors among Undergraduates of a Private University in Nigeria

**DOI:** 10.1155/2018/5439079

**Published:** 2018-12-20

**Authors:** Deborah Tolulope Esan, Ayodeji Akinwande Fasoro, Opeoluwa Esther Odesanya, Theophilus Olaide Esan, Elizabeth Funmilayo Ojo, Charles Oluwafemi Faeji

**Affiliations:** ^1^Department of Nursing, College of Medicine and Health Sciences, Afe Babalola University, Ado-Ekiti, Nigeria; ^2^Department of Public Health, College of Medicine and Health Sciences, Afe Babalola University, Ado-Ekiti, Nigeria; ^3^Depatment of E.N.T., Federal Teaching Hospital, Ido-Ekiti, Nigeria; ^4^Department of Medical Microbiology and Parasitology, College of Medicine and Health Sciences, Afe Babalola University, Ado-Ekiti, Nigeria

## Abstract

**Background:**

Self-medication is the use of drugs to treat self-diagnosed disorders or symptoms or the intermittent or continued use of prescribed drug for chronic or recurrent disease or symptoms, and it is mostly common in developing countries. This study therefore assessed the practice of self-medication among undergraduate students of a private university in Nigeria.

**Methods:**

The study employed a descriptive cross-sectional design. A pretested questionnaire was self-administered to 384 undergraduate students of the university. Data were analysed and summarised using descriptive and inferential statistics such as chi-squared and Fisher's exact tests.

**Results:**

Overall, 297 (81.8%) undergraduate students practiced self-medication. About 71% of the students had used analgesic, antibiotics (10.5%), and antimalarial drugs (33%) without prescription within one month prior to the survey. The most commonly used drug for self-medication was paracetamol (75.1%). Furthermore, self-medication was found to be significantly associated with age (*p*=0.021), gender (*p* < 0.001), college (*p*=0.025), and year of study (*p*=0.004). Some of the reasons why undergraduate students practiced self-medication were because of the unfriendly attitude of health care workers (27.7%), lack of time to go to school clinic (26.7%), school clinic is too far from hostel (15.3%), and drugs prescribed in the school clinic do not improve health condition (15.3%).

**Conclusion:**

Majority of the students attributed the practice of self-medication to unfriendly attitude of health care workers in the university clinic.

## 1. Introduction

Self-medication has been defined as the use of medication (modern and/or traditional) for self-treatment without consulting a physician either for diagnosis, prescription, or surveillance of treatment [1]. It involves obtaining medication without prescription and taking medicines on advice of and from friends and relatives. Self-medication is common in both developed and developing countries but higher in developing countries, due to wider increase of drug availability without prescription [2]. Self-medication increases the possibility of drug abuse [3] and drug dependency. It also masks the signs and symptoms of underlying diseases, hence complicating the problem, creating drug resistance, and delaying diagnosis [4, 5]. Self-medication has been reported to be on the rise globally [6]. The World Health Organization (WHO) emphasized that self-medication must be correctly taught and controlled in other to avoid drug-related issues such as antimicrobial resistance which is now a current problem worldwide particularly in developing countries where antibiotics are often available without a prescription [6].

Many studies have revealed that young adults are more vulnerable to the practice of self-medication due to their low perception of risk associated with the use of drugs, knowledge of drugs, easy access to Internet, wider media coverage on related health issues, ready access to drugs, level of education, and social status [7–9]. The practice of self-medication in general has been widely studied among populations of many countries in Africa, Asia, and Europe [7, 10, 11]. The sale of both over the counter (OTC) and prescription drugs by petty traders and roadside hawkers is very common in Nigeria [12]. In Nigeria, there are many unregistered patent medicine stores/pharmacies from which people purchase drugs from unknown sources [13]. Self-medication with both OTC and prescription drugs is very common in Nigeria. Previous studies have concentrated on general self-medication practices among the population [14] and health care workers [15]. This current study of self-medication practice among undergraduate students of a private university in Nigeria is very important as it seeks to provide insight into the health status of this educated group of individuals and with a clue to providing information regarding their state of health as well as OTC drug use. The understanding of self-medication practice and the reasons for it will enable different interventional strategies. The aim of this study was to assess self-medication practices among undergraduate students of a private university in Nigeria. It also aimed at estimating the prevalence of self-medication in the study population.

## 2. Methods

The study was conducted in all the five colleges of Afe Babalola University Ado-Ekiti (ABUAD), Ekiti State, Nigeria, from 13–30 June 2016. The university is a private university located in Ado-Ekiti, Ekiti State, Nigeria. This study employed a descriptive cross-sectional design, and undergraduate students were the study population. A sample size of 384 was calculated using Fisher's formula for a study using analytical study design with the level of confidence set at 95% and a precision of 0.05 [16]. The sample size was also adjusted for a 10% nonresponse. A multistage sampling procedure was employed to select respondents for this study. A cluster sampling was employed to select all the five colleges (Sciences, Law, Social and Management Sciences, Engineering, and Medicine and Health Sciences) in the first stage. A stratified random sampling was then used to select the departments to be sampled from a list of the departments in the five clusters (colleges). Nine departments were selected, and a proportionate allocation was done according to the size of each department. The multistage sampling method was employed to avoid selection bias. The course coordinators were approached to ascertain when the students will be in their respective lecture halls. The students were approached while in their lecture halls and recruited for the study. A total of 384 undergraduate students were sampled. The questionnaire was assessed for face and content validity by experts in the College of Medicine and Health Sciences of the University after it was pretested. The self-administered, semistructured, pretested questionnaires were printed on paper and were self-administered. The purpose of the study was explained to the respondents and their verbal and written consent to participate in the study were sought and obtained before the questionnaires were administered. The confidentiality of the participants was guaranteed, and they were informed that the data will be analysed at a group level in order to de-identify participants. The questionnaire was in five sections: section A contained the sociodemographic characteristics of respondents; section B contained questions on self-medication with analgesics; section C contained questions on self-medication with antibiotics and/or antimalarial; section D contained questions on source of commonly used drugs for self-medication; and section E contained questions on factors that influence the practice of self-medication. The questions assessed the self-medication practices in the past one month in order to minimise recall bias. Self-medication was assessed by asking if the respondent ever used analgesics, antimalarial drugs, and/or antibiotics in the past one month without prescription. Any drug from other sources except from a doctor or the school clinic was classified as self-medication. Data were analysed using Statistical Package for Social Sciences (SPSS) version 20. Data were presented in frequencies, percentages, means, and standard deviation with the aid of charts and tables. Bivariate analysis was done using chi-squared and Fisher's exact tests. The level of significance was set at *p* value < 0.05. To carry out the study, ethical clearance was obtained from the Ethics and Research Committee of Afe Babalola University, Ado-Ekiti.

## 3. Results

**Figure 1:**
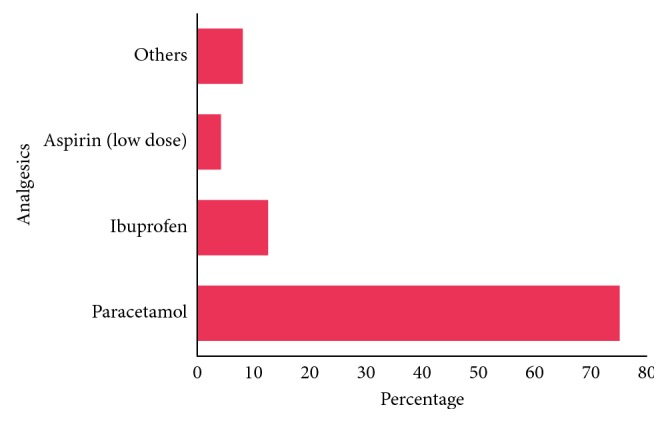
Commonly used analgesics for self-medication (*n*=258).

**Figure 2:**
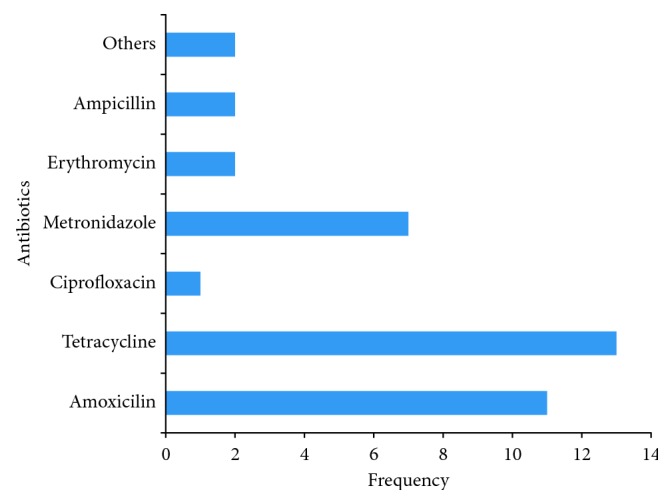
Antibiotic use for self-medication among respondents (*n*=38).

The sociodemographic characteristics of the respondents show that majority (62.8%) were females, between ages 19–23 years (63.3%), Christians (90.6%), and Yoruba (45.2%) as shown in Table 1. Approximately, 82% of the respondents admitted to self-medication practice. About 11% and 71.1% have used antibiotics and analgesics, respectively, in the past one month. Cough was the most common condition (3.6%) needing antibiotic use in the study population (Table 2); others were sore throat (1.9%) and gastroenteritis (1.9%). Paracetamol (75.1%) and ibuprofen (12.6%) were the mostly used analgesics for self-medication (Figure 1) while tetracycline (34.2%), amoxicillin (28.9%), and metronidazole (18.4%) were the mostly used antibiotics by the respondents for self-medication (Figure 2).

**Figure 3:**
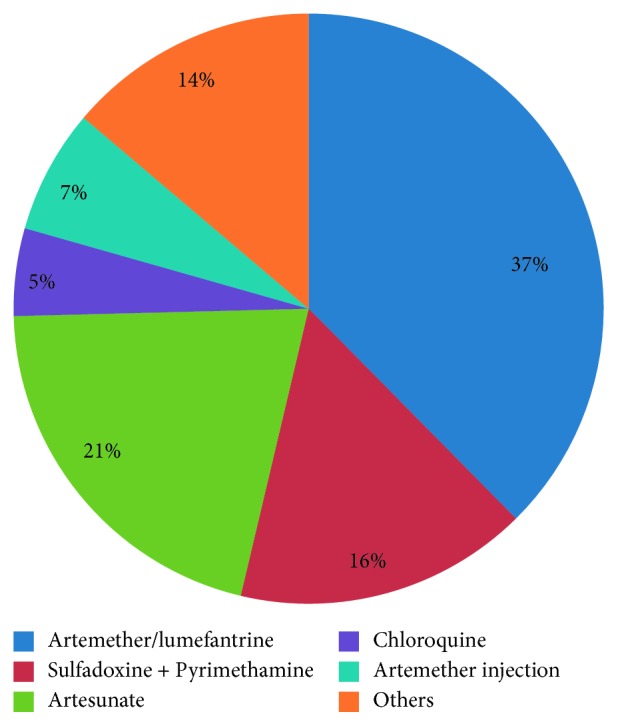
Commonly used antimalarial drugs for self-medication (*n*=120).

**Figure 4:**
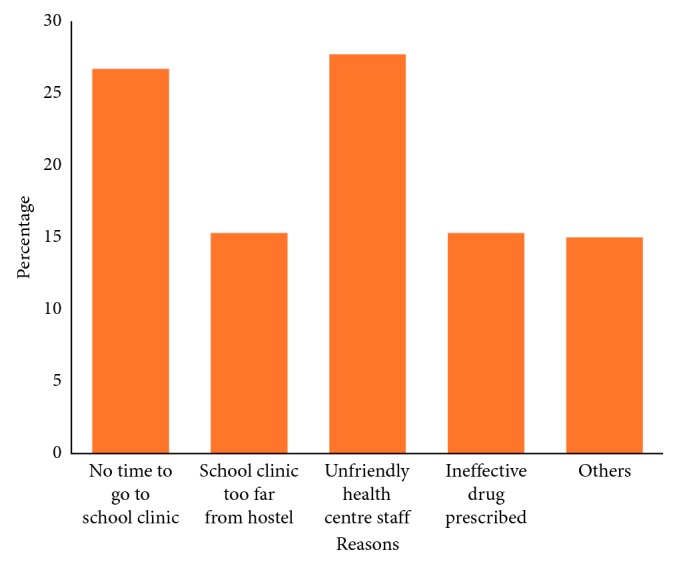
Reasons for practicing self-medication (*n*=297).

Out of 120 undergraduate students that practice self-medication with antimalarial, 37% of the undergraduate students recently used artemether/lumefantrine, artesunate (21%) and sulfadoxine + pyrimethamine (16%) for malaria treatment without the doctor's prescription (Figure 3). Some of the reasons why undergraduate students practiced self-medication were because of the unfriendly attitude of health care workers (27.7%), lack of time to go to school clinic (26.7%), school clinic is too far from hostel (15.3%), and drugs prescribed in the school clinic do not improve health condition (15.3%) (Figure 4). Self-medication practices was found to be significantly associated with age (*p*=0.021), gender (*p* < 0.001), college (*p*=0.025), and year of study (*p*=0.004) as seen in Table 3.

## 4. Discussion

A total of 363 valid responses were obtained, giving a response rate of 94.5%. This response rate was achieved probably due to the fact that the school being a private university disallows students from leaving the school premises until the end of the semester and occasionally when there is a religious holiday.

### 4.1. Prevalence of Self-Medication

The findings of this study showed that the prevalence of self-medication was 81.8% among the undergraduate students of this university. This prevalence is considerably high, however similar to 88% reported among students in Gujarat [17]; 87% and 88.2% among students in North India [18, 19] and 92% among students in South India [20]; 100% among students in Bangladesh [21]; 98% among students in Palestine [22]; 86.4% among students in Brazil [23]; and 91.4% in south-west Nigeria [24]. The prevalence reported in this study is higher than those reported in some other studies [25–28]. The differences could be as a result of the discipline of the students surveyed, country's drug laws, or the effectiveness of the drug regulating agencies of the countries where the studies were conducted. It is believed that students in medicine and other health sciences tend to self-medicate themselves than other students from other disciplines. This study also revealed that the prevalence of self-medication increased as the year of study increased. Similar result was reported among university students in south-west Nigeria [24]. The College of Law reported the highest prevalence (91.1%) of self-medication, followed by College of Medicine and Health Sciences (85.2%). One would have expected that students from the College of Medicine and Health Sciences would have taken the lead. This may be expected due to the fact that they are more knowledgeable about different ailments and drugs used in their treatments.

The practice of self-medication was higher among females (88.2%) than males (70.5%). This was found to be statistically significant (*p* < 0.001). This is similar to some other studies [17, 22, 24, 28], which identified female students as fundamental elements in the use of OTC drugs. In this study, as shown in Table 2, self-medication was found to be significantly associated with age (*p*=0.021), gender (*p* < 0.001), college (*p*=0.025), and year of study (*p*=0.004).

### 4.2. Major Drugs Used for Self-Medication

Prior to the conduct of the study, more than half (71.1%) of the respondents had used analgesic without the doctor's prescription in the last one month. Paracetamol (75.1%) was the mostly used drug among the students. Similar reports were found out in other studies [22, 28]. This could be because many believed paracetamol to be a nontoxic drug that can be used at any time, irrespective of the dosage without having any side effect.

Tetracycline (34.2%), amoxicillin (28.9%), and metronidazole (18.4%) were the mostly used antibiotics. Other studies found out that ampicillin and amoxicillin were the mostly self-medicated drugs among students [17, 26, 27]. Seventeen (44.7%) out of the 38 users of antibiotics got the antibiotics from their hostels, home, and other sources. This is in contrast with many studies that reported the major sources to be from pharmacies, patent medicine stores, friends, and families [24, 25]. The findings of this study may simply be because there are no pharmacy stores in the university campus where students can purchase OTC drugs. The most common conditions for which antibiotics were used among the 38 respondents were cough (34.2%), sore throat (18.4%), and gastroenteritis (18.4%). Self-medication practices with antibiotics in some studies were mostly reported to be for urinary tract infection [24], sore throat [23, 24, 27], gastrointestinal ailments [20], and cough [17, 23, 28].

One third (33%) of the students reported using antimalarial drugs without prescription. Artemether/lumefantrine (37%), artesunate (21%), and sulfadoxine + pyrimethamine (16%) were the mostly used antimalarial drugs. Self-medicated antimalarial use was also reported to be prevalent in some studies among tertiary institution students [24, 25].

### 4.3. Factors Promoting Practice of Self-Medication

We found out that the most common factors that led to self-medication among students were attributed to unfriendly attitude of health care workers at the school clinic (27.7%), busy schedule of students that resulted into lack of time to visit the clinic (26.8%), distance of the school clinic to the hostel (15.3%), and perceived inefficacy of prescribed drug (15.3%). Various studies reported different reasons for engaging self-medication. These include knowledge about the disease/treatment [25], previous experience [25], availability of medications [19], mild diseases [18, 25], affordability [18, 19, 26], and to save time [18, 19, 26, 29]. These reasons however are subject to the environment and study populations where the studies were carried out.

## 5. Conclusions

This study concluded that majority of the respondents practiced self-medication and this was majorly attributed to unfriendly attitude of health care workers in the university clinic. Commonly used drugs were paracetamol, artemether/lumefantrine, and tetracycline. Self-medication may not be harmful on its own, but it poses a great threat when OTC and prescription drugs become abused. Health education on self-medication should be introduced into the undergraduate curriculum so as to enlighten the students on the risks and benefits of self-medication. The university should also create a friendly atmosphere in the university clinic so as to encourage the students to visit the clinic anytime they feel symptoms of any disease.

## 6. Limitations

One of the limitations of this study was that the study employed a cross-sectional study design and as such causal relationships between variables cannot be established. Also, the analyses were based on self-report with the possibility of over and under reporting. The results of this study cannot be generalised to a larger population of university students in the state or the country.

## Figures and Tables

**Table 1:** Sociodemographic characteristics of respondents.

Characteristics	Frequency	Percentage
Gender		
** **Male	135	37.2
** **Female	228	62.8

Age (years)		
** **Less than 19	123	33.9
** **19–23	230	63.3
** **24–28	10	2.8

College		
** **Engineering	63	17.4
** **Law	45	12.4
** **Medicine and health sciences	61	16.8
** **Sciences	51	14.0
** **Social and management sciences	143	39.4

Year of study		
** **First	68	18.7
** **Second	103	28.4
** **Third	73	20.1
** **Fourth	103	28.4
** **Fifth	16	4.4

Religion		
** **Christian	329	90.6
** **Muslim	31	8.6
** **Others	3	0.8

Ethnicity		
** **Hausa	17	4.7
** **Igbo	82	22.6
** **Others	100	27.5
** **Yoruba	164	45.2

**Table 2:** Use of antibiotics and analgesics among respondents.

	Frequency (*N*=363)	Percentage
Use of antibiotics in the past one month		
** **Yes	38	10.5
** **No	325	89.5

Source of supply of antibiotics		
** **Doctor	9	2.5
** **School clinic	12	3.3
** **Hostel	3	0.8
** **From home	9	2.5
** **Others	5	1.4
** **Missing data/not applicable	325	89.5

Conditions which antibiotics were used		
** **Sore throat	7	1.9
** **Bronchitis	5	1.4
** **Gastroenteritis	7	1.9
** **Urinary tract infection	4	1.1
** **Cough	13	3.6
** **Others	2	0.6
** **Missing data/not applicable	325	89.5

Use of analgesics in the past one month		
** **Yes	258	71.1
** **No	105	28.9

Conditions analgesic was used for (multiple answers allowed)		
** **Headache	167	46.0
** **Stomachache	26	7.2
** **Body pain	54	14.9
** **Muscle pain	15	4.1
** **Dysmenorrhoea	43	11.8
** **Fever	11	3.0
** **Cough/cold	11	3.0
** **Arthritis pain	4	1.1
** **Others	15	4.1
** **Missing data/not applicable	105	28.9

**Table 3:** Association between sociodemographic characteristics and self-medication practice.

Sociodemographic characteristics	Self-medication		No self-medication		*χ* ^2^/Fisher's exact	*p* value
	*n*	%	*n*	%		
Overall	297	81.8	66	18.2		

Gender						
** **Male	96	71.1	39	28.9	16.564	<0.001^*∗*^
** **Female	201	88.2	27	11.8		

Age (years)						
** **Less than 19	92	74.8	31	25.2	8.007	0.015^*∗*^
** **19–23	198	86.1	32	13.9		
** **24–28	7	70.0	3	30.0		

Ethnicity						
** **Hausa	13	76.5	4	23.5	0.546	0.910
** **Igbo	66	80.5	16	19.5		
** **Others	83	83.0	17	17.0		
** **Yoruba	135	82.3	29	17.7		

Religion						
** **Christianity	267	81.2	62	18.8	2.386	0.283
** **Islam	28	90.3	3	9.7		
** **Others	2	66.7	1	33.3		

Academic year						
** **First	53	77.9	15	22.1	15.503	0.004^*∗*^
** **Second	74	71.8	29	28.2		
** **Third	62	84.9	11	15.1		
** **Fourth	92	89.3	11	10.7		
** **Fifth	16	100.0	0	0.0		

College						
** **Engineering	48	76.2	15	23.8	11.153	0.025^*∗*^
** **Law	41	91.1	4	8.9		
** **Medicine and health sciences	52	85.2	9	14.8		
** **Sciences	0.35	68.6	16	31.4		
** **Social and management sciences	121	84.6	22	15.4		

^*∗*^Significant at *p* value <0.05.

## Data Availability

The data used to support the findings of this study are available from the corresponding author upon request.

## Abbreviations

WHO: World Health Organization
OTC: Over the counter
ABUAD: Afe Babalola University, Ado-Ekiti
SPSS: Statistical Package for Social Sciences.

## References

[1] Hughes C. M., McElnay J. C., Fleming G. F. (2001). Benefits and risks of self medication. *Drug Safety*.

[2] Klemenc-Ketis Z., Hladnik Z., Kersnik J. (2010). Self-medication among healthcare and non-healthcare students at university of Ljubljana, Slovenia. *Medical Principles and Practice*.

[3] McCabe S. E., Teter C. J., Boyd C. J. (2005). Illicit use of prescription pain medication among college students. *Drug and Alcohol Dependence*.

[4] Bauchner H., Wise P. H. (2000). Antibiotics without prescription: “bacterial or medical resistance”?. *The Lancet*.

[5] Calabresi P., Cupini L. M. (2005). Medication-overuse headache: similarities with drug addiction. *Trends in Pharmacological Sciences*.

[6] World Health Organization (1998). The role of the pharmacist in self-care and self-medication. http://www.who.int/medicines/library/dap/whodap-98-13/who-dap-98-13.pdf.

[7] Flaiti M. A., Badi K. A., Hakami W. O., Khan S. A. (2014). Evaluation of self-medication practices in acute diseases among university students in Oman. *Journal of Acute Disease*.

[8] Di Muzio M., De Vito C., Tartaglini D., Villari P. (2017). Knowledge, behaviours, training and attitudes of nurses during preparation and administration of intravenous medications in intensive care units (ICU). A multicenter Italian study. *Applied Nursing Research*.

[9] Simone E. D., Tartaglini D., Fiorini S., Petriglieri S., Plocco C., Muzio M. D. (2016). Medication errors in intensive care units: nurses’ training needs. *Emergency Nurse*.

[10] Yousef A. M., Al-Bakri A. G., Bustanji Y., Wazaify M. (2007). Self-medication patterns in Amman, Jordan. *Pharmacy World and Science*.

[11] Awad A. I., Eltayeb I. B., Capps P. A. (2006). Self-medication practices in Khartoum state, Sudan. *European Journal of Clinical Pharmacology*.

[12] Adelusi-Adeluyi J. Drug distribution: challenges and effects on the Nigerian society.

[13] Erhun W. O., Adeola M. A. (1995). A study of the distribution of fake drugs in Ogun State Nigeria. *Nigerian Journal of Pharmaceutical Research*.

[14] Afolabi A. (2008). Factors influencing the pattern of self-medication in an adult Nigerian population. *Annals of African Medicine*.

[15] Bamgboye E. A., Amoran O. E., Yusuf O. B. (2006). Self medication practices among workers in a tertiary hospital in Nigeria. *African Journal of Medicine and Medical Sciences*.

[16] Aday L. A., Cornelius L. J. (2006). *Designing and Conducting Health Surveys: A Comprehensive Guide*.

[17] Pateh M. M., Singh U., Sapre C., Salvi K., Shah A., Vasoya B. (2013). Self-medication practices among college students: a cross sectional study in Gujarat. *National Journal of Medical Research*.

[18] Verma R. K., Mohan L., Pandey M. (2010). Evaluation of self-medication among professional students in North India: proper statutory drug control must be implemented. *Asian Journal of Pharmaceutical and Clinical Research*.

[19] Goel D. D. (2013). Self-medication patterns among nursing students in North India. *IOSR Journal of Dental and Medical Sciences*.

[20] Badiger S., Kundapur R., Jain A. (2012). Self-medication patterns among medical students in South India. *Australasian Medical Journal*.

[21] Alam N., Saffoon N., Uddin R. (2015). Self-medication among medical and pharmacy students in Bangladesh. *BMC Research Notes*.

[22] Sawalha A. F. (2007). Assessment of self-medication practice among university students in Palestine: therapeutic and toxicity implications. *IUG Journal of Natural Studies*.

[23] Corrêa da Silva M. G., Soares M. C. F., Muccillo-Baisch A. L. (2012). Self-medication in university students from the city of Rio Grande, Brazil. *BMC Public Health*.

[24] Osemene K. P., Lamikanra A. (2012). A study of the prevalence of self-medication practice among university students in southwestern Nigeria. *Tropical Journal of Pharmaceutical Research*.

[25] Auta A., Shalkur D., Omale S., Abiodun A. H. (2012). Medicine knowledge and self-medication practice among students. *African Journal of Pharmaceutical Research and Development*.

[26] Donkor E., Tetteh-Quarcoo P., Nartey P., Agyeman I. (2012). Self-medication practices with antibiotics among tertiary level students in Accra, Ghana: a cross-sectional study. *International Journal of Environmental Research and Public Health*.

[27] Fadare J. O., Tamuno I. (2011). Antibiotic self-medication among university medical undergraduates in Northern Nigeria. *Journal of Public Health and Epidemiology*.

[28] Gutema G. B., Gadisa D. A., Kidanemariam Z. A. (2011). Self-medication practices among health sciences students: the case of Mekelle university. *Journal of Applied Pharmaceutical Science*.

[29] Pirzadeh A., Mostafavi F. (2014). Self-medication among students in isfahan university of medical sciences based on health belief model. *Journal of Education and Health Promotion*.

